# Principle Study of Head Meridian Acupoint Massage to Stress Release via Grey Data Model Analysis

**DOI:** 10.1155/2016/4943204

**Published:** 2016-01-19

**Authors:** Ya-Ting Lee

**Affiliations:** Department of Beauty Science, National Taichung University of Science and Technology, No. 193, Sec. 1, San-Min Road, Taichung 40343, Taiwan

## Abstract

This paper presents the scientific study of the effectiveness and action principle of head meridian acupoint massage by applying the grey data model analysis approach. First, the head massage procedure for massaging the important head meridian acupuncture points including Taiyang, Fengfu, Tianzhu, Fengqi, and Jianjing is formulated in a standard manner. Second, the status of the autonomic nervous system of each subject is evaluated by using the heart rate variability analyzer before and after the head massage following four weeks. Afterward, the physiological factors of autonomic nerves are quantitatively analyzed by using the grey data modeling theory. The grey data analysis can point out that the status of autonomic nervous system is greatly improved after the massage. The order change of the grey relationship weighting of physiological factors shows the action principle of the sympathetic and parasympathetic nerves when performing head massage. In other words, the grey data model is able to distinguish the detailed interaction of the autonomic nervous system and the head meridian acupoint massage. Thus, the stress relaxing effect of massaging head meridian acupoints is proved, which is lacked in literature. The results can be a reference principle for massage health care in practice.

## 1. Introduction

In the last twenty years, the meridian theory is becoming an important therapy in complementary medicine. Different from traditional medicinal treatments, the meridian therapy takes acupuncture massage to palliate the symptoms of patients [1–3]. The traditional Chinese medicine has pointed out that the meridian acupuncture massage has a curative effect to headaches, dizziness, stiff neck, shoulder and back pain, stomachache, and so forth. As a result, many researches were proposed to understand the effects and mechanisms of the meridian therapy [4–11]. For worthwhile examples, the stress can be relaxed by the back massage [12]; dysmenorrheal and lumbar spondylolisthesis can be palliated by massaging corresponding acupunctures [13, 14]; the meridian massage can reduce weight and control physiological index for simple obesity patients [15]; and so on. Among these benefits of meridian therapy, the effect of reducing stress has received much attention in the contemporary age because most of people are subject to high stress in daily life [12, 16, 17]. It is true that the stress physically and cognitively affects the body, where the physical impacts include muscle tension, shallow and frequent breathing, tachycardia, high blood pressure, and the secretion of adrenalin and the cognitive impacts include difficulty concentrating and memory problems. If the stress is not coped with appropriately or the body and mind are not properly adjusted, then chronic stress will induce various physical and psychological responses, for example, diseases of the nervous, endocrine, immune, and reproductive systems. Although authors in [16, 17] observe the stress release after body massage, the evaluation on stress change is only according to blood pressure, heart rate, and feeling of the patient. On the other hand, since stress is body's reaction to events, thoughts, or emotions, the heart rate variability (HRV) [18] can show psychological change of the stress even simple deep breathing exercises. There are many researches that indicate that the HRV reflects the status of autonomic nervous system (cf. [19]). Accordingly, the HRV is measured for evaluating the effect of meridian massage therapy in researches [19–22] for more scientific and accurate study. However, these works [19–22] cannot find the interaction model from incomplete data statistics of the physiological change during the process of the massage therapy. Moreover, a lot of experiments are required for accurately studying the effect of the meridian massage. In other words, interaction quantitative analysis is lacked in current literature. In addition, although Chinese meridian theory [1] claims that massaging acupuncture points on head and shoulders are related to improvement of the body stress, very few works study the related field on the head massage [23]. This is because the meridian acupunctures on the head are more complex than the human body. Also, the characteristic and quantitative analysis of head meridian acupuncture massage is lacked in literature. No integrated method can offer explanation of the impact and physiological changes due to head acupuncture point massage so far. The effects in most of massage therapies are still measured qualitatively through feeling feedback from subjects. Thus, more scientific analysis is required for studying the head meridian massage therapy.

From the pioneering work of Professor Deng [24], grey system theory has been rigorously utilized in a variety of fields including engineering science [25, 26], medicine [27], data analysis [28, 29], and grey relational analysis [30]. Since the grey system theory can minimize the randomness of the data series and the interference from the random information by applying the grey generating, the numerical data do not need to satisfy classical distribution. In other words, the grey system theory is able to solve the analysis problem of incomplete information data and realize many significant and effective applications. In addition, the grey system theory is able to construct the data model of complex systems and estimate the relationship between the input and output factors. Here the characteristic of the data system can be clarified from calculating the relationship weighting of the grey data model. Moreover, the grey relationship weighting is obtained from the least square estimation method which is a kind of optimal linear regression approach; that is, the grey model can emulate the data system in an optimized manner even if less data samples are considered. In contrast, traditional regression analysis or statistics commonly require a very large amount of data to obtain a correct analyzed result of system behavioral data. As a result, the grey model theory is suitably applied on the characteristic analysis of the head meridian acupoint massage which accompanies complex and uncertain physiological relation.

Motivated by the above, this study investigates the effect of massaging acupuncture points on the head and uses the grey system theory for assistant data analysis. First, the study method of the head meridian acupoint massage is introduced. Then, since the HRV is able to reflect the status of autonomic nervous system [18–22], quantitative features of the HRV of test subjects are observed before and after the head meridian acupoint massage. Here the power spectral analysis of the HRV data in 5 minutes is performed to present the autonomic nerve activity [31, 32] and transformed to the important physiological factors: physiological stress index (PSI), very low frequency (VLF) power, low frequency (LF) power, high frequency (HF) power, total power (TP), and LF/HF ratio. Total of 45 middle-aged women in Taiwan join this experiment for four weeks, while all the test subjects are with long-term work stress and high PSI before the massage. Furthermore, the grey GM(0, *N*) model of the data system is constructed to find the relationship between the head massage and each physiological factor of HRV (i.e., status of the autonomic nervous system). As a result, the grey relationship weighting points out the characteristic that head meridian massage relaxes the stress and improves autonomic nervous system function. The detailed physiological action principle of the head massage is understood from integrating the statistic and grey modeling analysis. In addition, the effective model of the head meridian massage can be clarified without complex mathematic calculation and enormous amount of data.

In this paper, we firstly formulate the study method of head meridian massage in Section 2. In Section 3, the grey GM(0, *N*) model is presented to data analysis. In Section 4, the analyzed and discussed results are presented. Finally, we make some conclusions and suggestions for the further research in Section 5.

## 2. Method of Head Massage

To perform the scientific study of the head massage, a standard procedure of massaging head meridian acupuncture points is given in the following.

### 2.1. Location of Important Meridian Acupuncture Points

First, let us introduce five important meridian acupuncture points on the head and the shoulders which belong to the extra channel, Du meridian, urinary bladder meridian, and gall bladder meridian, respectively.

*(i) EX-HN5 (Taiyang)*. Taiyang acupoint is an extraordinary acupuncture point. The EX-HN5 acupoint is in the depression about one fingerbreadth behind the midpoint of a line connecting the lateral end of the eyebrow and the outer canthus of the eye. The action and effects are dependent on migraine headaches, dizziness, and eye issues.

*(ii) UB10 (Tianzhu).* Tianzhu acupoint is on urinary bladder meridian which will benefit neck issues, stiffness, and occipital headache. This point is located about 5 cm lateral from the midline in the depression on the lateral aspect of the trapezius muscle. Massaging this point may improve memory.

*(iii) Du16 (Fengfu).* Du16 is an important point at the Du meridian. The point is located superiorly about 3 cm on the edge of the thumb joint which is horizontally placed on the hairline at the midline of the nape of the neck; that is, it is directly below the occipital protuberance on the posterior midline of the head. This is the main point for wind, whether exterior or interior, particularly affecting the head and neck.

*(iv) GB20 (Fengqi).* Fengqi acupoint belongs to the gall bladder meridian which helps digest food and stores bile produced by the liver. This point is located laterally to the sternomastoid and the trapezius muscles in the back below the occipital bone and on a level with the earlobe and Fengfu. The point is indicated for headaches, heaviness of the head, soreness of the eyes and the neck, stiff neck, insomnia, and hangovers. This acupuncture point is usually massaged together with EX-HN5.

*(v) GB21 (Jianjing).* GB21 is also on the gall bladder meridian and is the meeting point of the foot Taiyang urinary bladder, the Du, and the Yang linking meridians. This acupoint is on the midway between the spinous process of cervical vertebra and the acromion process at the highest point of the trapezius muscle. Massaging this point may effectively relieve a stiff neck, neck pain, and shoulder and back pain.

Although some actions and effects of these acupuncture points are claimed in Chinese medicine, scientific analysis is lacked in related researches. Thus, the massaging effect to autonomic nerve activity will be estimated and analyzed in this study.

### 2.2. Experimental Method

The scientific experimental procedure of the massage analysis is described in the following. First, each subject takes a rest before the testing. Then, the physiological data of the autonomic nervous system is measured before the head massage. Afterward, the massage of meridian acupuncture points is performed in turn from EX-HN5, GB20, UB10, and Du16 to GB21 for the subject. Finally, the physiological data of the autonomic nervous system is measured again after the massage. The same test procedure continues for four weeks for all subjects. In detail, the experimental setting and the massage method are given below.

#### 2.2.1. Environmental Setting

In our experiments, a quiet room is chosen as the test site to reduce the environmental affection. The room temperature is maintained at 26 degrees Celsius. The test is performed at a specific time from 18:00 to 21:00 in everyday to avoid interference of the heart rate variability for the time difference.

#### 2.2.2. Measurement of Autonomic Nervous Indices

Since the heart rate variability (HRV) is affected by respiration, blood vessels, endocrine, and emotions, the measurement of heart rate variability can reflect the mutual influence between the sympathetic nerve, the parasympathetic nerve, and the cardiac sinus node. This means that we can clarify the balance and the activity of autonomic nerves through measuring and analyzing the heart rate variability [18–22]. Thus, the heart rate variability is measured by a heart rate variability analyzer, SA-3000P, made by Medicore Co., Ltd. [33]. This equipment measures the heart rate variability of the subject in five minutes, while the quantitative values are analyzed by using Fast Fourier Transfer and Power Spectral Density techniques. Then, the autonomic nervous indices are obtained including physiological stress index (PSI), total power (TP), very low frequency (VLF) power, low frequency (LF) power, high frequency (HF) power, and LF/HF ratio. These physiological indices are related to emotion and stress of subjects [8–10]. Before the testing of the heart rate variability, some important rules should be obeyed as follows:

- Remove every metal object from the body before the heart rate variability is measured.
- Do not wear nail polish, which may affect measurement.
- Avoid medication or stimulating drinks, such as coffee, tea, or alcohol, and avoid hunger or overeating.
- Rest 10 minutes before testing.
- Avoid any movement when the measurement is taken to avoid interfering with the accuracy.

The autonomic nervous indices are measured by the following procedure:

- Hold the sensory clamp on the left index or middle finger.
- Sit properly by resting the back against the chair and close the eyes.
- Place both hands on the lap and relax.
- Wait until the EKG wave has been stabilized on the screen about 5 minutes and then record the data of the autonomic nerve indices.

#### 2.2.3. Steps for Head Massaging

The standard massage procedure is performed by starting from the acupuncture point EX-HN5 to GB20 in the nape, to Du16 and UB10 on the head, and then to GB21 between the shoulder and the neck. The detailed process is given in the following steps.

**Step 1 .**

**Figure 1:**
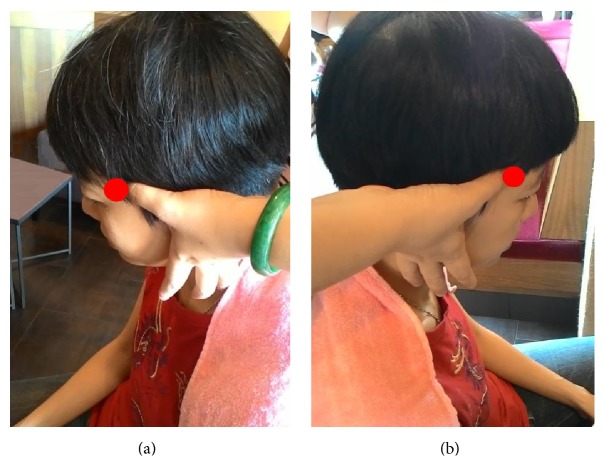
EX-HN5 (Taiyang) acupuncture points on (a) left side and (b) right side of the head.

First, knead EX-HN5 (Taiyang) acupuncture point. The head massage starts from the EX-HN5 acupoint with thumbs or middle-fingers of two hands as per the demonstration shown in Figure 1. The gesture is clockwise iteratively in four eight-beats (about 1 minute).

**Step 2 .**

Slightly massage the top of the scalp by using the fingertips of forefinger, middle-finger, ring finger, and pinky finger of two hands in four eight-beats. Knock the top of the scalp by a fixed tempo and a comfortable strength in four eight-beats.

**Step 3 .**

**Figure 2:**
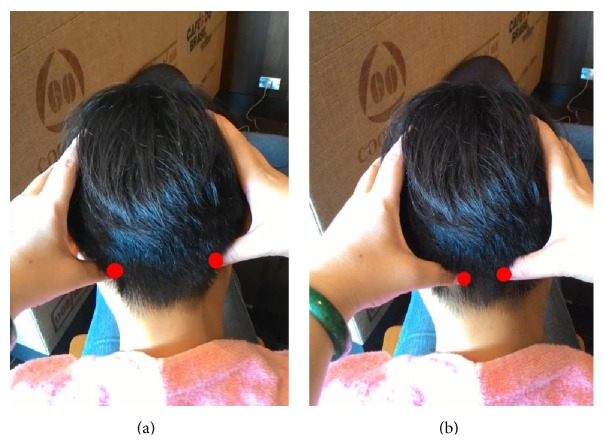
(a) GB20 (Fengqi) acupuncture points on the head; (b) UB10 (Tianzhu) acupuncture points on the head.

Knead GB20 (Fengqi) acupuncture point on the nape. The GB20 acupuncture point is massaged by using two thumbs as per the demonstration shown in Figure 2(a). The massage gesture is clockwise iteratively in four eight-beats.

**Step 4 .**

Knead UB10 (Tianzhu) acupuncture point. By using the two thumbs and placing the little and index finger around the corner of the eye, UB10 is clockwise massaged in iterative four eight-beats as per the demonstration shown in Figure 2(b). The weight of the head is used to press on the thumb to massage UB10.

**Step 5 .**

**Figure 3:**
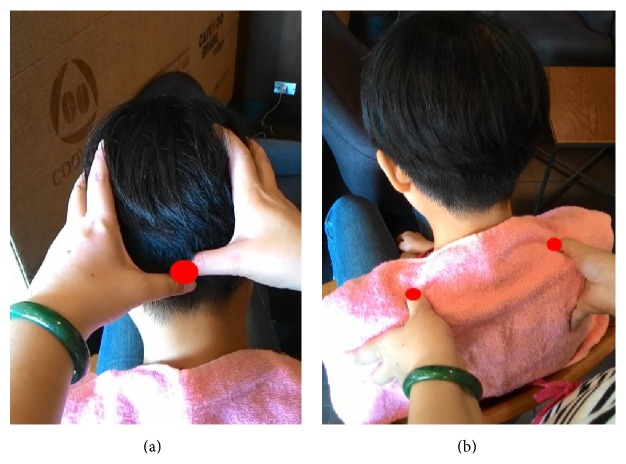
(a) Du16 (Fengfu) acupuncture point on the head; (b) GB21 (Jianjing) acupuncture points on shoulders.

Knead Du16 (Fengfu) acupuncture point. By using the two thumbs, Du16 is clockwisely massaged in iterative four eight-beats as per the demonstration shown in Figure 3(a).

**Step 6 .**

Relax the neck. We knead the neck from Fengqi to the shoulders in slight force strength. The action is iterative from top to down by using the two thumbs in four eight-beats.

**Step 7 .**

Knead GB21 (Jianjing) acupuncture point on shoulders. Grasp the shoulders by the fingers and press the Jianjing acupoint by the thumb, while Jianjing is clockwisely massaged in iterative four eight-beats as per the demonstration shown in Figure 3(b).

The massage process is performed by one massagist who has perfect hand skill. The acupuncture points can be correctly located, while the massage strength is properly controlled. The massage process costs about 10 minutes. After taking a rest, the status of the autonomic nervous system is evaluated by using the heart rate variability analyzer. The same experimental approach as the above is done one time per week and continued for four weeks.

In this study, total of 45 women with long-term work stress are chosen as the test subjects, who are with the averaged 40 years of age (from 25 to 55 years old). All subjects are without symptoms or histories of cardiovascular or other diseases. To demonstrate the stress relaxing effect, recruited participants satisfy the criterion either usually feeling chronic stress/fatigue or having a large PSI (above 50) before the massage. Indeed, the averaged PSI of these screened subjects is larger than 50 before the head meridian acupoint massage (i.e., this fact is shown in Section 4). After acupuncture point massaging experiments applying the above stated method on all subjects in four weeks, the data obtained from the measurement of the heart rate variability analyzer is shown in Table 1. Table 1 lists the changes of the activity of autonomic nerves before and after massage in each week, while showing significant lowering of the PSI after massaging.

*Remark*. Since the purpose of this study is to analyze the physiological changes of the autonomic nervous system due to the acupuncture point massage, any possible factors which affect the result should be avoided. Indeed, the subject's lifestyle, sleeping patterns, and emotions are required to control in a regular manner, while extravagant consumption of coffee, tea, food, and medication should be avoided before the experiment. On the other hand, the massagist does her best in localizing the acupuncture points and massaging strength, such that the result is correct.

## 3. Grey Theory of Data Analysis

**Figure 4:**
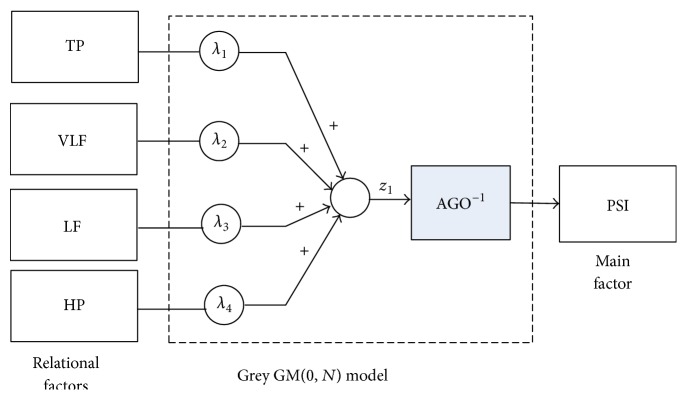
The configuration of the grey GM(0, *N*) model for data analysis.

To further quantize the effect of head massage, this section applies the grey model GM(0, *N*) to analyze the measured data of the physiological indices of autonomic nerves. In grey system theory, the main function of GM(0, *N*) model is one of the methods to carry out the relationship weighting calculation among the discrete sequences of measured data. To analyze the relationship of the physiological factors of the head acupoint massage, the grey model structure is constructed as illustrated in Figure 4. The physiological stress index (PSI) is taken as the major sequence factor, while the total power (TP), very low frequency (VLF) power, low frequency (LF) power, and high frequency (HF) power are taken as the influencing sequence factors. The grey GM(0, *N*) model will describe the relationship between the influencing sequence factors and the major sequence factor (i.e., the relationship model between the pressure potency and the other measured physiological factors). Furthermore, the relationship weighting can represent the characteristic of the resultant physiological situation for the acupoint massage. This means that the change of weighting factors of the grey GM(0, *N*) model after performing acupoint massage can point out the effectiveness and action rules of the head acupoint massage. To this end, the grey GM(0, *N*) modeling is introduced and applied to identify the relationship weighting factors in the following.

### 3.1. Grey *GM*(0, *N*) Model

First, let us denote the data sequence of the main factor (PSI) as *x*_0_^(0)^(*k*); that is,

(1) $$\begin{array}{c} x_{0}^{\left(0\right)}=\left(\;x_{0}^{\left(0\right)}\left(\;1\;\right),x_{0}^{\left(0\right)}\left(\;2\;\right),\ldots ,x_{0}^{\left(0\right)}\left(\;L\;\right)\;\right), \end{array}$$

where *L* is the sequence length of the measured data and the superscript “(0)” means the original data. The data sequences of influence factors, which are TP, VLF, LF, and HF, are, respectively, defined as follows:

(2) $$\begin{array}{c} x_{1}^{\left(0\right)}=\left(\;x_{1}^{\left(0\right)}\left(\;1\;\right),x_{1}^{\left(0\right)}\left(\;2\;\right),\ldots ,x_{1}^{\left(0\right)}\left(\;L\;\right)\;\right),\\ x_{2}^{\left(0\right)}=\left(\;x_{2}^{\left(0\right)}\left(\;1\;\right),x_{2}^{\left(0\right)}\left(\;2\;\right),\ldots ,x_{2}^{\left(0\right)}\left(\;L\;\right)\;\right),\\ \vdots \\ x_{N-1}^{\left(0\right)}=\left(\;x_{N-1}^{\left(0\right)}\left(\;1\;\right),x_{N-1}^{\left(0\right)}\left(\;2\;\right),\ldots ,x_{N-1}^{\left(0\right)}\left(\;L\;\right)\;\right), \end{array}$$

where *N* is the total sequence number of data and *N* = 5 in this case. Since the measured data is not dynamic sequence, the grey zero-order GM(0, *N*) model is used for our static data analysis. Without loss of generality, the GM(0, *N*) model is represented in the following form:

(3) $$\begin{array}{c} ax_{0}^{\left(1\right)}\left(\;k\;\right)=\overset{N-1}{\underset{j=1}{\sum }}b_{j}x_{j}^{\left(1\right)}\left(\;k\;\right), \end{array}$$

where *a* and *b*_*j*_ are some coefficients; *k* = 1, 2, …, *L* is the data index; *x*_*j*_^(1)^(*k*), for *j* = 0,1,…, *N* − 1, are the first-order accumulative generation operations (1-AGO) of the original sequences *x*_*j*_^(0)^(*k*) and are defined as follows:

(4) $$\begin{array}{c} x_{j}^{\left(1\right)}\left(\;k\;\right)=\overset{k}{\underset{g=1}{\sum }}x_{j}^{\left(0\right)}\left(\;g\;\right). \end{array}$$

This means that the resultant 1-AGO sequence is

(5) $$\begin{array}{c} x_{j}^{\left(1\right)}=\left(\;\overset{1}{\underset{g=1}{\sum }}x_{j}^{\left(0\right)}\left(\;g\;\right),\overset{2}{\underset{g=1}{\sum }}x_{i}^{\left(0\right)}\left(\;g\;\right),\ldots ,\overset{L}{\underset{g=1}{\sum }}x_{i}^{\left(0\right)}\left(\;g\;\right)\;\right). \end{array}$$

Obviously, the accumulative generation operation is applied to convert the sequences to strict monotonic increasing sequences, such that the randomness is reduced and the smoothness of the sequence is increased. From (3), the grey GM(0, *N*) model is a special type of multiple regressive modeling that is distinct from traditional ones. Also, the GM(0, *N*) model is a special case of the grey GM(*h*, *N*) model without derivatives (i.e., *h* = 0).

Furthermore, there exist some parameters *b*_*j*_′ such that the grey model (3) is equivalent to the following noise-free model:

(6) $$\begin{array}{c} az_{0}^{\left(1\right)}\left(\;k\;\right)=\overset{N-1}{\underset{j=1}{\sum }}b_{j}^{\prime }x_{j}^{\left(1\right)}\left(\;k\;\right), \end{array}$$

where *z*_0_^(1)^(*k*) is called the *k*th background values for the grey differential equation and is generated from averaging the adjacent data sequence of *x*_0_^(1)^(*k*) as follows:

(7) $$\begin{array}{c} z_{0}^{\left(1\right)}\left(\;k\;\right)=\frac{1}{2}\left(\;x_{0}^{\left(1\right)}\left(\;k\;\right)+x_{0}^{\left(1\right)}\left(\;k-1\;\right)\;\right) \end{array}$$

for *k* = 2,3,…, *L*. Then, the GM(0, *N*) model can be expressed by one variable through the following zero-order grey differential equation:

(8) az01k∑j=1N−1bj′xj1k=b1′x11k+b2′x21k+⋯+bN−1′xN−11k,

where the coefficients *a* and *b*_1_′, *b*_2_′,…, *b*_*N*−1_′ are called the grey developing and grey input coefficients, respectively. After applying the 1-AGO and the averaged adjacent data sequence, we obtain the overall grey equations (for *N* = 5):

(9) $$\begin{array}{c} az_{0}^{\left(1\right)}\left(\;2\;\right)=b_{1}^{\prime }x_{1}^{\left(1\right)}\left(\;2\;\right)+\cdots +b_{N-1}^{\prime }x_{N-1}^{\left(1\right)}\left(\;2\;\right),\\ az_{0}^{\left(1\right)}\left(\;3\;\right)=b_{1}^{\prime }x_{1}^{\left(1\right)}\left(\;3\;\right)+\cdots +b_{N-1}^{\prime }x_{N-1}^{\left(1\right)}\left(\;3\;\right),\\ \vdots \\ az_{0}^{\left(1\right)}\left(\;L\;\right)=b_{1}^{\prime }x_{1}^{\left(1\right)}\left(\;L\;\right)+\cdots +b_{N-1}^{\prime }x_{N-1}^{\left(1\right)}\left(\;L\;\right). \end{array}$$

Afterward, by dividing *a* on both sides of the above equations and rearranging the GM(0, *N*) model in a matrix form, we obtain

(10) z012z013⋮z01L=x112x212⋯xN−112x113x213⋯xN−113⋮⋮⋯⋮x11Lx21L⋯xN−11Lλ1λ2⋮λN−1,

where *λ*_*j*_ = *b*_*j*_′/*a* for *j* = 0,1,…, *N* − 1. Notice that the developing coefficient *a* ≠ 0 and *λ*_*j*_ is the characteristic weighting for the relation from the influence sequences *x*_1_^(0)^,…, *x*_*N*−1_^(0)^ to the major sequence factor *x*_0_^(0)^. To solve the characteristic weighting, let us denote the following vector and matrix:

(11) z^=z012z013⋮z01L=0.5x012+x0110.5x013+x012⋮0.5x01L+x01L−1,λ=λ1λ2⋮λN−1,X=x112⋯xN−112x113⋯xN−113⋮⋯⋮x11L⋯xN−11L.

Then, the grey GM(0, *N*) model (10) is expressed in a standard linear regressive form as follows:

(12) $$\begin{array}{c} \hat{z}=X\lambda . \end{array}$$

Thus, by using the least square estimation method, the weighting coefficients can be obtained as follows:

(13) $$\begin{array}{c} \lambda ={\left(\;X^{T}X\;\right)}^{-1}X^{T}\hat{z}. \end{array}$$

Since the coefficients *λ*_1_,…, *λ*_*N*−1_ carry the intrinsic information contained in the data sequences, the coefficients *λ*_1_,…, *λ*_*N*−1_ can indicate the relationship between the major sequence (PSI) and the other influence factors (TP, VLF, LF, and HF). When the data property is changed, the relationship weighting is changed. In other words, the relationship weighting *λ*_1_,…, *λ*_*N*−1_ is able to show the level of the physiological state. Therefore, we will observe the change of the relationship weighting of the grey GM(0, *N*) model to distinguish the effect of the head massage.

### 3.2. Data Analysis Calculation

Based on the measured data in Table 1, we take the mean of the data in 4 weeks as our analysis subjects and omit the dependent factor (LF/HF). Tables 2 and 3, respectively, show the averaged data of 4 weeks before and after the head meridian acupoint massage for each subject. According to the grey GM(0, *N*) model in the above subsection, we let the grey factors be *x*_0_ = PSI, *x*_1_ = TP, *x*_2_ = VLF, *x*_3_ = LF, and *x*_4_ = HF. This means that the physiological data sequences before the head acupoint massage are defined from Table 2 as follows:

(14) x00=38.09,82.06,25.86,…,37.41,85.53,52.78,x10=1797.85,683.10,1685.74,…,1340.39,930.42,1218.26,x20=770.11,297.60,524.09,…,441.80,316.54,422.38,x30=670.26,179.24,371.14,…,352.99,339.96,471.98,x40=357.11,205.64,790.71,…,545.59,273.74,323.90.

Note that the length of each data sequence is *L* = 45. On the other hand, the physiological data sequences after the head acupoint massage are defined according to Table 3 as follows:

(15) x00=27.26,57.65,22.30,…,30.16,58.49,34.91,x10=2145.09,1028.64,2049.12,…,1690.68,1257.92,1749.37,x20=604.52,289.92,524.03,…,463.38,452.31,548.38,x30=750.0,365.22,568.10,…,439.46,327.37,539.62,x40=803.2,360.98,932.14,…,787.83,478.24,661.38.

By substituting the above data sequences into (10), the relationship weighting for each factor is obtained from (13). Note here that the calculation only runs one time for the data because the relationship weighting is solved according to the least square estimation method which is a kind of optimal linear regression approach. To assure the correctness, the numerical calculation is performed by using Matlab software. The program allows any number of subjects and complex calculation. Then, the results of the grey model analysis are obtained in Table 4.

## 4. Results and Discussion

After continuing the massaging of head and shoulder acupuncture points in four weeks, the changes of the autonomic nervous system are analyzed by using the grey GM(0, *N*) model theory. Table 4 shows the results of the grey model weighting before and after performing the head massage. Indeed, each physiological index has almost the same grey relationship weighting (to be about 55.3672) prior to acting on the head acupoint massage. After massaging from the acupuncture point EX-HN5 to acupuncture point GB21 through four weeks, the relationship weighting for the grey GM(0, *N*) data modeling is obviously reduced to small values about 0.831. Since there exists a very large change of the relationship weighting of the grey data modeling, the head meridian acupoint massage has huge influence on the autonomic nervous system of subjects. This is one powerful proof for the effect of head meridian acupoint massage. Furthermore, since the influencing level is proportional to the relationship weighting of the grey data model, the importance of the influencing factors is changed before and after acting on the head massage from Table 4. The influencing level before the head massage is sequentially

(16) $$\begin{array}{c} LF>VLF>HF>TP, \end{array}$$

while the influencing level after the head massage is follows:

(17) $$\begin{array}{c} HF>LF>TP>VLF. \end{array}$$

The fact that the influencing level of HF becomes larger indicates that the activity of parasympathetic nerves is enhanced. Since LF and VLF factors represent the activity of sympathetic nerves, the change of the influencing level between LF and VLF factors is trivial. As a result, the activity of parasympathetic nerves is enhanced to balance the autonomic nervous system after performing the head massage at meridian acupoints including DU16 and GB21. In addition, the influencing level of TP becomes larger after the head massage, so that the capability of subjects for coping with stress is increased. Thus, from the above quantitative description, the acupuncture point massage links a strong relationship between the physiological changes of the autonomic nerves, parasympathetic nerves, and stress regulation.

To run cross validation, different numbers of data samples are considered in the grey GM(0, *N*) model analysis. Four cases of the grey relationship calculation are performed with total 45, 30, 20, and 15 randomly chosen data samples, respectively, from Tables 2 and 3. Then, the results are obtained as shown in Table 5. From Table 5, they have the same property of large change of the relationship weighting after the head massage; that is, the effect of the head massage is indicated. Moreover, after the head massage, the rank of the grey relationship weighting is the same as HF > LF > TP > VLF for the four cases. This implies that the action principle of the HRV physiological factors can be found even if less data samples are used for the grey GM(0, *N*) model. This result shows that the grey model method has an assistant capability of data analysis for traditional methods.

**Figure 5:**
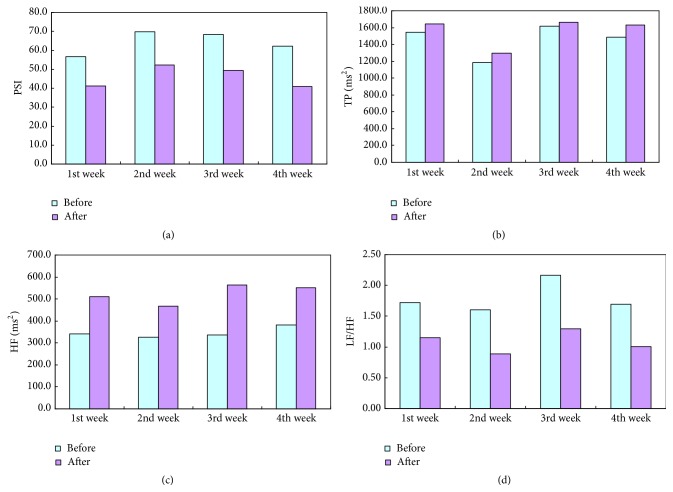
For all subjects in 4 weeks: (a) the averaged PSI, (b) the averaged TP, (c) the averaged HF, and (d) the averaged LF/HF.

On the other hand, the physiological change can be also observed from averaging each factor data of all subjects per week; that is, Figure 5 illustrates the effect of head meridian massage during four weeks. Obviously, the averaged PSI value of subjects is reduced after performing head meridian massage (cf.  Figure 5(a)). The relaxation effect of PSI is averaged 28.6% with respect to the pretest PSI from 56.6 to 69.8. This effect matches the big change of the relationship weighting in the grey data models before and after head massage. In other words, the stress is able to be reduced by massaging acupuncture points EX-HN5 through GB21. In terms of total power (TP), subjects with an index lower than the normal range of 1000~2000 would feel tiredness and weakness, which is mostly caused by long-term fatigue and chronic diseases. From Figure 5(b), the total power value has been increased every time of the head massage. This means that the head massage can relax fatigue and increase spirit of people.

In addition, the averaged HF value of the heart rate variability after the head massage is higher than that of the value before the massage shown in Figure 5(c), where this phenomenon satisfies the influence level change of HF in the grey GM(0, *N*) model. This means that the activity of parasympathetic nerves is increased. Meanwhile, Figure 5(d) illustrates that the LF/HF ratio has significant reduction below 2, where the normal range of LF/HF is from 1 to 2. The decrease of LF/HF ratio implies that the sympathetic nerve comes down. In other words, the sympathetic and parasympathetic nerves would get more balance. The stress relief of massaging head meridian acupoints is achieved by regulating the activity balance of sympathetic and parasympathetic nerves. Thus, the statistic results match the grey data analysis. The most important thing is that massaging acupuncture points on the head and shoulder can regulate cardiac autonomic nervous functions, relieve psychological stress, and improve cardiovascular activities. Therefore, the above result can be a reference for stress relief and health care in massaging meridian acupuncture points of Chinese medicine.

## 5. Conclusions

In this paper, the effect of massaging acupuncture points on the head and shoulders has been explored by observing the change of autonomic nervous system and using grey GM(0, *N*) model analysis. From the grey GM(0, *N*) model analysis, the experimental data for massaging the acupuncture points from EX-HN5 to GB21 in four weeks is characterized by the grey relationship weighting. The principle of head massage acting on the autonomic nervous system is observed according to the change of the grey relationship weighting. As a result, the head massage has significant benefits to the autonomic nervous system function. The physiological stress is relaxed and the activity of sympathetic and parasympathetic nerves is regulated to more balance. The result offers clear explanation of the physiological changes for head meridian acupoint massage which was not scientifically proved in previous studies on health care. Consequently, quantitative features of the head meridian acupoint massage are obtained from this study.

## Figures and Tables

**Table 1:** The HRV indices before and after massage.

Subject	Factor	Before 1st week	After 1st week	Before 2nd week	After 2nd week	Before 3rd week	After 3rd week	Before 4th week	After 4th week
01	PSI	36.96	30.17	64.89	44.04	22.13	16.36	28.39	18.49
	TP (ms^2^)	1631.1	1950.8	672.9	2038.6	2856.3	2364.6	2031.1	2226.4
	VLF (ms^2^)	600.0	724.1	353.5	775.4	1632.1	473.1	494.8	445.5
	LF (ms^2^)	777.2	759.3	201.5	570.4	923.9	899.4	778.4	770.9
	HF (ms^2^)	253.0	567.4	117.9	693.2	300.2	1042.1	757.3	910.0
	LF/HF	3.07	1.34	1.71	0.82	3.08	0.86	1.03	0.85

02	PSI	95.54	62.60	34.68	29.54	120.66	82.47	77.37	56.00
	TP (ms^2^)	557.6	602.6	811.4	1668.9	752.5	520.3	610.9	1322.7
	VLF (ms^2^)	254.1	125.3	191.0	346.8	440.3	166.8	305.0	520.8
	LF (ms^2^)	200.6	276.4	206.5	720.4	120.0	127.5	189.9	336.6
	HF (ms^2^)	101.9	201.0	413.0	601.7	191.7	226.0	116.0	415.3
	LF/HF	1.97	1.38	0.50	1.20	0.63	0.56	1.64	0.81

03	PSI	22.22	20.98	36.80	23.09	22.31	22.60	22.10	22.52
	TP (ms^2^)	1316.0	1531.0	1155.9	2406.9	1941.8	1779.0	2329.3	2479.6
	VLF (ms^2^)	335.0	336.1	181.5	650.3	399.4	309.8	1180.4	799.8
	LF (ms^2^)	249.9	537.7	325.5	748.7	489.0	545.2	420.2	440.8
	HF (ms^2^)	731.9	557.8	648.8	1007.8	1053.3	924.0	728.7	1239.0
	LF/HF	0.34	0.96	0.50	0.74	0.46	0.59	0.58	0.36

04	PSI	37.23	28.04	43.62	24.76	34.09	20.64	55.92	28.02
	TP (ms^2^)	1276.8	1607.5	1454.1	1606.7	1242.5	1355.5	1597.9	1815.8
	VLF (ms^2^)	571.4	457.6	668.5	562.1	599.3	353.9	407.7	229.0
	LF (ms^2^)	117.1	548.8	271.7	223.0	302.2	270.1	588.2	311.3
	HF (ms^2^)	588.3	601.1	513.9	821.7	341.1	731.4	602.0	1275.6
	LF/HF	0.20	0.91	0.53	0.27	0.89	0.37	0.98	0.24

05	PSI	76.44	58.32	53.07	18.71	99.99	73.00	23.52	34.90
	TP (ms^2^)	743.0	1098.5	1490.5	2343.0	2109.5	1178.5	402.0	2295.0
	VLF (ms^2^)	172.5	495.0	298.0	623.0	1261.5	346.5	132.5	864.0
	LF (ms^2^)	384.0	250.5	540.5	718.0	641.5	336.5	146.5	903.5
	HF (ms^2^)	186.0	303.0	650.0	1002.0	206.0	596.0	120.0	526.5
	LF/HF	2.06	0.83	0.83	0.72	3.11	0.56	1.22	1.72

06	PSI	23.77	21.95	25.39	13.73	64.64	50.25	63.06	34.63
	TP (ms^2^)	801.6	2800.9	2353.7	3300.8	736.9	809.5	734.6	2192.4
	VLF (ms^2^)	290.9	958.6	307.6	1715.7	55.3	393.1	380.3	731.9
	LF (ms^2^)	155.8	776.8	1232.2	877.7	535.8	173.0	226.0	644.0
	HF (ms^2^)	354.9	1065.4	813.9	807.4	145.8	243.4	128.3	816.5
	LF/HF	0.44	0.73	1.51	1.09	3.68	0.71	1.76	0.79

07	PSI	67.01	52.41	70.26	58.00	55.82	36.22	78.76	56.15
	TP (ms^2^)	563.9	1423.0	433.9	727.1	535.7	1085.4	785.5	1036.3
	VLF (ms^2^)	421.9	737.1	235.0	275.1	138.7	247.4	517.5	359.7
	LF (ms^2^)	108.6	357.6	152.6	213.3	237.1	371.0	155.0	191.5
	HF (ms^2^)	33.3	428.2	46.3	238.7	159.9	467.1	112.9	485.1
	LF/HF	3.26	0.84	3.30	0.89	1.48	0.79	1.37	0.39

08	PSI	33.87	27.54	26.11	14.68	44.89	36.04	49.31	28.90
	TP (ms^2^)	1135.9	2463.7	1758.8	3405.9	1240.2	1638.3	1165.4	2049.6
	VLF (ms^2^)	400.4	819.9	466.3	1406.3	447.3	348.1	474.0	633.5
	LF (ms^2^)	409.6	902.2	783.6	971.7	605.3	711.4	489.9	506.4
	HF (ms^2^)	326.0	741.6	508.9	1027.9	187.6	628.8	201.5	809.5
	LF/HF	1.26	1.22	1.54	0.95	3.23	1.13	2.43	0.63

09	PSI	43.97	33.13	26.83	15.62	25.15	21.82	35.56	23.18
	TP (ms^2^)	1470.3	2126.6	1163.9	3511.1	1743.5	2467.2	1596.2	1906.8
	VLF (ms^2^)	509.9	681.1	625.0	1196.9	839.3	303.1	567.7	835.3
	LF (ms^2^)	663.3	1027.6	335.0	1065.7	674.9	1149.8	753.8	568.8
	HF (ms^2^)	297.0	417.9	203.9	1248.5	229.4	1014.3	274.6	502.8
	LF/HF	2.23	2.46	1.64	0.85	2.94	1.13	2.75	1.13

10	PSI	33.89	28.67	44.97	39.13	58.07	52.17	30.00	25.00
	TP (ms^2^)	1072.8	2636.7	980.1	816.2	1187.0	606.7	1422.4	1727.5
	VLF (ms^2^)	204.9	562.5	471.2	165.6	657.7	159.6	369.7	586.5
	LF (ms^2^)	314.9	1349.9	379.1	423.7	166.0	144.0	263.1	528.1
	HF (ms^2^)	553.0	724.3	129.8	226.9	363.3	303.0	789.6	612.8
	LF/HF	0.57	1.86	2.92	1.87	0.46	0.48	0.33	0.86

11	PSI	29.95	27.22	102.95	72.45	19.11	10.90	21.21	13.80
	TP (ms^2^)	1792.0	1775.0	182.0	566.0	3969.0	2262.0	2466.0	2546.0
	VLF (ms^2^)	690.0	767.0	82.0	354.0	2425.0	643.0	422.0	1110.0
	LF (ms^2^)	891.0	491.0	68.0	74.0	1173.0	547.0	803.0	973.0
	HF (ms^2^)	209.0	517.0	32.0	138.0	371.0	1070.0	1240.0	462.0
	LF/HF	4.26	0.95	2.13	0.54	3.16	0.51	0.65	2.11

12	PSI	35.47	30.42	146.11	86.07	183.25	112.77	114.19	86.99
	TP (ms^2^)	550.0	701.0	135.0	389.0	318.0	434.0	655.0	918.0
	VLF (ms^2^)	177.0	131.0	37.0	85.0	223.0	174.0	264.5	555.0
	LF (ms^2^)	98.0	91.0	22.0	129.0	74.0	111.0	239.5	145.0
	HF (ms^2^)	275.0	479.0	74.0	175.0	20.0	149.0	151.1	217.0
	LF/HF	0.36	0.19	0.30	0.74	3.70	0.74	1.59	0.67

13	PSI	49.93	44.19	180.87	175.52	84.94	23.63	47.05	39.86
	TP (ms^2^)	1304.0	1332.0	250.0	298.0	515.0	875.0	804.0	2798.0
	VLF (ms^2^)	263.0	636.0	98.0	50.0	174.0	136.0	265.0	1038.0
	LF (ms^2^)	700.0	427.0	110.0	126.0	278.0	463.0	293.0	916.0
	HF (ms^2^)	340.0	269.0	41.0	122.0	63.0	276.0	246.0	844.0
	LF/HF	2.06	1.59	2.68	1.03	4.41	1.68	1.19	1.09

14	PSI	12.95	10.53	22.47	19.27	16.15	12.70	33.70	16.80
	TP (ms^2^)	9216.0	2068.0	3063.0	1069.0	3455.0	1281.0	2661.0	1246.0
	VLF (ms^2^)	5636.0	836.0	919.0	239.0	939.0	353.0	869.0	952.0
	LF (ms^2^)	3178.0	562.0	1617.0	410.0	1617.0	253.0	1336.0	100.0
	HF (ms^2^)	402.0	670.0	527.0	418.0	899.0	675.0	456.0	194.0
	LF/HF	7.91	0.84	3.07	0.98	1.80	0.37	2.93	0.52

15	PSI	50.45	40.54	57.61	48.57	56.94	44.19	52.92	40.57
	TP (ms^2^)	818.4	2029.9	707.0	771.7	861.4	846.1	949.1	1381.9
	VLF (ms^2^)	313.4	649.8	353.1	220.3	398.2	203.5	315.5	473.1
	LF (ms^2^)	211.8	853.7	265.9	318.5	201.6	257.5	292.2	359.8
	HF (ms^2^)	293.2	576.3	88.0	232.8	261.6	385.0	341.4	549.0
	LF/HF	0.72	1.48	3.02	1.37	0.77	0.67	0.86	0.66

16	PSI	159.07	105.77	106.19	77.98	164.15	151.82	172.88	81.30
	TP (ms^2^)	184.0	565.0	423.0	406.0	328.0	234.0	293.0	617.0
	VLF (ms^2^)	68.0	157.0	180.0	173.0	92.0	23.0	63.0	298.0
	LF (ms^2^)	45.0	238.0	136.0	147.0	143.0	179.0	93.0	220.0
	HF (ms^2^)	71.0	170.0	107.0	86.0	93.0	32.0	137.0	99.0
	LF/HF	0.63	1.40	1.27	1.71	1.54	5.59	0.68	2.22

17	PSI	102.34	64.00	73.56	55.73	92.04	83.18	98.90	53.49
	TP (ms^2^)	723.0	1663.0	1112.5	717.0	1381.5	3176.5	1418.5	1169.5
	VLF (ms^2^)	301.0	542.0	362.0	245.0	241.0	1090.0	418.5	518.5
	LF (ms^2^)	121.5	652.0	310.0	218.0	657.0	1625.0	573.5	402.0
	HF (ms^2^)	300.5	469.0	441.5	254.0	483.5	461.5	426.5	249.0
	LF/HF	0.40	1.39	0.70	0.86	1.36	3.52	1.34	1.61

18	PSI	39.72	31.77	86.47	50.85	104.20	67.29	74.88	55.09
	TP (ms^2^)	1010.1	1413.8	648.4	1950.0	1030.3	1450.6	1125.6	1411.9
	VLF (ms^2^)	343.5	406.1	331.0	640.9	531.1	238.6	416.1	695.2
	LF (ms^2^)	380.7	559.3	178.5	597.3	374.4	630.4	496.7	356.9
	HF (ms^2^)	286.0	448.4	138.9	711.7	124.7	581.6	212.9	359.9
	LF/HF	1.33	1.25	1.28	0.84	3.00	1.08	2.17	0.99

19	PSI	73.97	43.12	57.24	44.61	55.98	48.86	61.91	39.59
	TP (ms^2^)	992.5	2212.0	1457.8	872.5	1981.7	4647.8	1981.3	1445.8
	VLF (ms^2^)	417.5	734.5	453.0	281.0	315.5	1623.5	596.3	628.8
	LF (ms^2^)	159.8	859.0	397.0	253.5	914.0	2348.0	813.8	493.0
	HF (ms^2^)	415.3	618.5	607.8	338.0	752.2	676.3	571.3	324.0
	LF/HF	0.38	1.39	0.65	0.75	1.22	3.47	1.42	1.52

20	PSI	144.89	95.33	98.03	72.42	146.12	134.66	106.34	74.35
	TP (ms^2^)	318.8	839.5	595.7	483.8	591.4	969.6	563.4	854.0
	VLF (ms^2^)	126.3	253.3	225.5	191.0	129.3	289.8	261.0	353.1
	LF (ms^2^)	64.1	341.5	179.5	164.8	271.5	540.5	193.4	265.5
	HF (ms^2^)	128.4	244.8	190.6	128.0	190.6	139.4	109.0	235.4
	LF/HF	0.50	1.40	0.94	1.29	1.42	3.88	1.77	1.13

21	PSI	42.70	37.30	163.49	130.79	134.09	68.20	80.62	63.43
	TP (ms^2^)	926.5	1016.5	192.5	343.5	416.5	654.5	729.5	1857.5
	VLF (ms^2^)	220.0	383.5	67.5	67.5	198.5	155.0	264.7	796.5
	LF (ms^2^)	399.0	259.0	64.5	127.5	176.0	287.0	266.2	530.5
	HF (ms^2^)	307.5	374.0	60.5	148.5	42.0	212.5	198.5	530.5
	LF/HF	1.30	0.69	1.07	0.86	4.19	1.35	1.39	1.00

22	PSI	31.44	27.36	101.67	97.39	50.55	18.16	40.37	28.33
	TP (ms^2^)	5259.5	1700.0	1656.0	682.5	1985.0	1078.0	1732.5	2022.0
	VLF (ms^2^)	2949.5	736.0	508.5	144.5	556.5	244.5	567.0	995.0
	LF (ms^2^)	1939.0	494.5	863.5	268.0	947.5	358.0	814.5	508.0
	HF (ms^2^)	371.0	469.5	284.0	270.0	481.0	475.5	351.0	519.0
	LF/HF	5.23	1.05	3.04	0.99	1.97	0.75	2.32	0.98

23	PSI	32.71	28.82	124.53	79.26	101.18	61.83	67.70	50.40
	TP (ms^2^)	1171.0	1238.0	157.5	477.5	2143.0	1347.0	1560.0	1731.0
	VLF (ms^2^)	433.5	449.0	59.5	219.5	1324.0	408.5	343.2	832.5
	LF (ms^2^)	494.5	291.0	45.0	101.5	623.5	329.0	521.2	559.0
	HF (ms^2^)	242.0	498.0	53.0	156.5	195.5	609.5	695.5	339.5
	LF/HF	2.04	0.58	0.85	0.65	3.19	0.54	1.12	1.65

24	PSI	34.13	26.83	141.91	123.98	52.02	17.26	39.94	35.70
	TP (ms^2^)	1635.0	2672.0	215.5	432.0	2242.0	1567.5	1546.5	1553.5
	VLF (ms^2^)	343.5	1074.0	90.0	202.0	1299.5	389.5	476.5	701.5
	LF (ms^2^)	548.0	944.5	89.0	100.0	725.5	505.0	795.5	459.0
	HF (ms^2^)	743.0	653.0	36.5	130.0	217.0	673.0	274.5	393.0
	LF/HF	0.74	1.45	2.44	0.77	3.34	0.75	2.90	1.17

25	PSI	73.94	51.90	24.21	20.47	84.29	52.67	99.70	42.00
	TP (ms^2^)	1658.0	1081.5	4883.0	1384.5	1598.0	728.0	1886.0	857.5
	VLF (ms^2^)	566.7	753.5	2906.5	483.5	478.0	162.0	581.0	263.5
	LF (ms^2^)	787.7	122.5	1638.0	326.5	819.5	269.5	845.5	182.0
	HF (ms^2^)	303.5	205.5	338.5	574.5	300.5	296.5	459.5	412.0
	LF/HF	2.26	0.60	4.84	0.57	2.73	0.91	1.84	0.44

26	PSI	44.61	36.70	46.28	40.30	46.31	29.65	16.85	39.33
	TP (ms^2^)	940.4	1477.3	1187.8	1253.1	845.8	1746.2	1330.5	1758.0
	VLF (ms^2^)	378.5	536.6	317.2	292.5	160.1	448.8	434.5	579.8
	LF (ms^2^)	179.3	447.6	320.8	379.3	281.3	559.9	668.0	316.1
	HF (ms^2^)	382.6	493.0	549.8	581.3	404.4	737.4	228.0	862.1
	LF/HF	0.47	0.91	0.58	0.65	0.70	0.76	2.93	0.37

27	PSI	97.27	68.09	126.15	82.03	173.70	132.29	143.54	84.15
	TP (ms^2^)	367.0	633.0	278.0	397.5	323.0	334.0	474.0	767.0
	VLF (ms^2^)	122.5	144.0	108.5	129.0	157.5	98.5	163.7	426.5
	LF (ms^2^)	71.5	164.5	79.0	138.0	108.5	145.0	166.2	182.5
	HF (ms^2^)	173.0	324.5	90.5	130.5	56.5	90.5	144.0	158.0
	LF/HF	0.41	0.51	0.87	1.06	1.92	1.60	1.13	1.16

28	PSI	40.60	30.58	35.23	20.19	29.62	21.23	38.08	25.60
	TP (ms^2^)	1373.5	1867.0	1309.0	2558.9	1493.0	1911.3	1484.8	1861.3
	VLF (ms^2^)	540.6	569.3	646.7	879.5	719.3	328.5	554.2	532.1
	LF (ms^2^)	390.2	788.2	303.4	644.3	488.5	710.0	572.0	440.0
	HF (ms^2^)	442.7	509.5	358.9	1035.1	285.2	872.8	358.6	889.2
	LF/HF	0.88	1.55	0.85	0.62	1.71	0.81	1.81	0.49

29	PSI	28.83	25.31	35.18	26.43	61.35	41.00	26.03	20.04
	TP (ms^2^)	937.2	2718.8	1666.9	2058.5	961.9	708.1	2263.5	1722.3
	VLF (ms^2^)	247.9	760.6	389.4	940.6	356.5	276.4	743.6	517.1
	LF (ms^2^)	235.4	1063.4	805.7	650.7	350.9	158.5	987.0	398.9
	HF (ms^2^)	454.0	894.9	471.8	517.1	254.5	273.2	532.9	706.3
	LF/HF	0.52	1.19	1.71	1.26	1.38	0.58	1.85	0.56

30	PSI	88.49	53.40	127.21	115.62	76.13	54.10	72.97	46.68
	TP (ms^2^)	948.3	2025.8	681.3	507.5	1013.0	1497.5	1111.3	1983.8
	VLF (ms^2^)	207.5	613.0	230.0	147.5	282.0	589.0	341.8	778.3
	LF (ms^2^)	467.5	1044.0	210.0	172.0	410.8	539.5	433.3	659.0
	HF (ms^2^)	273.3	368.8	241.3	188.0	320.3	369.0	336.3	546.5
	LF/HF	1.71	2.83	0.87	0.91	1.28	1.46	1.29	1.21

31	PSI	72.12	65.10	56.17	43.06	71.82	47.71	84.80	42.10
	TP (ms^2^)	2154.9	1493.1	1915.7	815.3	4834.8	1591.0	1758.4	1069.6
	VLF (ms^2^)	552.8	454.8	595.0	224.0	2910.3	592.8	554.9	680.1
	LF (ms^2^)	1008.5	577.5	920.0	296.3	1630.6	503.5	834.6	205.5
	HF (ms^2^)	593.6	460.9	400.6	294.0	293.9	494.8	368.9	184.0
	LF/HF	1.70	1.25	2.30	1.01	5.55	1.02	2.26	1.12

32	PSI	29.72	24.51	32.97	23.68	35.45	21.86	47.11	25.27
	TP (ms^2^)	1296.8	1569.2	1697.9	1692.9	1199.2	1881.2	2242.1	2147.7
	VLF (ms^2^)	453.2	396.9	533.9	435.9	390.4	502.1	866.6	514.4
	LF (ms^2^)	183.5	543.2	380.4	384.1	313.8	509.4	714.3	376.0
	HF (ms^2^)	660.1	629.1	783.6	872.8	494.9	869.6	661.2	1257.3
	LF/HF	0.28	0.86	0.49	0.44	0.63	0.59	1.14	0.30

33	PSI	49.36	35.44	34.51	19.25	30.50	24.99	29.19	21.68
	TP (ms^2^)	989.7	1082.4	1903.9	2453.7	1039.2	2204.2	2067.5	2142.1
	VLF (ms^2^)	327.3	373.5	488.1	1138.9	431.1	708.1	733.6	600.0
	LF (ms^2^)	419.0	221.6	751.9	550.3	136.5	662.8	987.0	554.2
	HF (ms^2^)	243.4	487.4	663.9	814.5	471.6	833.3	346.9	987.9
	LF/HF	1.72	0.45	1.13	0.68	0.29	0.80	2.85	1.14

34	PSI	60.23	33.00	70.91	45.39	47.82	35.87	99.78	67.39
	TP (ms^2^)	636.3	947.4	1393.8	1614.4	1393.8	2063.9	661.8	1170.7
	VLF (ms^2^)	97.0	320.2	271.3	545.8	271.3	995.4	278.5	216.1
	LF (ms^2^)	386.4	272.0	692.4	417.7	692.4	545.5	250.5	499.2
	HF (ms^2^)	152.8	355.2	430.1	650.8	430.1	523.0	132.8	455.3
	LF/HF	2.53	0.77	1.61	0.64	1.61	1.04	1.89	1.10

35	PSI	40.48	29.02	48.54	39.66	48.54	36.81	55.49	42.77
	TP (ms^2^)	1139.6	1776.3	798.9	1471.6	798.9	2119.1	1017.1	1824.8
	VLF (ms^2^)	489.0	275.3	430.0	597.5	430.0	736.0	465.9	709.1
	LF (ms^2^)	456.0	760.4	243.8	380.1	243.8	639.5	386.0	692.6
	HF (ms^2^)	194.6	740.7	125.1	493.9	125.1	743.6	165.2	423.0
	LF/HF	2.34	1.03	1.95	0.77	1.95	0.86	2.34	1.64

36	PSI	41.61	36.99	35.90	24.09	35.90	27.38	26.65	21.90
	TP (ms^2^)	1465.3	1536.9	1072.0	1817.2	1072.0	1963.6	3801.6	1962.1
	VLF (ms^2^)	748.5	231.4	548.1	710.9	548.1	681.2	1967.9	525.4
	LF (ms^2^)	420.4	646.9	357.1	548.4	357.1	544.7	1392.6	664.3
	HF (ms^2^)	296.4	658.7	166.8	557.8	166.8	737.7	441.0	772.4
	LF/HF	1.42	0.98	2.14	0.98	2.14	0.74	3.50	1.14

37	PSI	45.61	22.24	40.92	33.49	19.93	14.53	24.92	25.68
	TP (ms^2^)	1262.0	2761.0	1802.0	1028.0	2435.0	6119.0	2544.0	1722.0
	VLF (ms^2^)	534.0	927.0	544.0	317.0	390.0	2157.0	774.0	739.0
	LF (ms^2^)	198.0	1066.0	484.0	289.0	1171.0	3071.0	1054.0	584.0
	HF (ms^2^)	530.0	768.0	774.0	422.0	874.0	891.0	716.0	399.0
	LF/HF	0.37	1.39	0.63	0.68	1.34	3.45	1.47	1.46

38	PSI	29.28	16.38	31.70	26.38	18.04	13.62	29.31	21.24
	TP (ms^2^)	5239.0	2414.5	2432.5	1047.5	2945.0	3700.0	2602.5	1484.0
	VLF (ms^2^)	3085.0	881.5	731.5	278.0	664.5	1255.0	821.5	845.5
	LF (ms^2^)	1688.0	814.0	1050.5	349.5	1394.0	1662.0	1195.0	342.0
	HF (ms^2^)	466.0	719.0	650.5	420.0	886.5	783.0	586.0	296.5
	LF/HF	3.62	1.13	1.61	0.83	1.57	2.12	2.04	1.15

39	PSI	130.70	84.89	89.87	66.86	128.09	117.50	135.89	67.39
	TP (ms^2^)	453.5	1114.0	768.3	561.5	854.8	1705.3	855.8	893.3
	VLF (ms^2^)	184.5	349.5	271.0	209.0	166.5	556.5	240.8	408.3
	LF (ms^2^)	83.3	445.0	223.0	182.5	400.0	902.0	333.3	311.0
	HF (ms^2^)	185.8	319.5	274.3	170.0	288.3	246.8	281.8	174.0
	LF/HF	0.45	1.39	0.81	1.07	1.39	3.66	1.18	1.79

40	PSI	86.01	58.15	64.33	48.62	90.15	82.26	101.08	49.05
	TP (ms^2^)	4700.0	1316.5	1743.0	736.5	1891.5	757.5	2766.0	931.5
	VLF (ms^2^)	2852.0	496.5	549.5	206.0	515.5	188.0	1574.0	625.0
	LF (ms^2^)	1611.5	400.0	876.5	278.5	880.0	216.0	890.5	160.0
	HF (ms^2^)	236.5	420.0	317.0	252.0	496.0	353.5	301.5	146.5
	LF/HF	6.81	0.95	2.76	1.11	1.77	0.61	2.95	1.09

41	PSI	53.96	40.24	86.60	65.22	37.46	23.56	44.96	28.43
	TP (ms^2^)	1856.6	2130.8	307.9	646.6	2252.4	2072.4	889.1	1520.5
	VLF (ms^2^)	467.8	732.0	158.5	314.5	1281.9	645.2	369.0	500.7
	LF (ms^2^)	808.5	922.3	110.3	143.7	705.1	459.0	269.6	320.6
	HF (ms^2^)	580.4	476.5	39.1	188.4	265.4	968.2	250.5	699.2
	LF/HF	1.19	1.60	2.82	0.76	2.66	0.47	1.08	0.46

42	PSI	52.12	40.22	56.94	41.38	27.28	23.76	60.78	42.08
	TP (ms^2^)	920.4	1565.2	944.0	1166.9	5340.6	2202.2	1600.4	1926.0
	VLF (ms^2^)	496.6	597.4	451.7	418.6	3192.2	633.2	501.3	594.1
	LF (ms^2^)	112.9	453.2	212.1	218.2	1799.2	604.5	636.8	451.6
	HF (ms^2^)	310.8	514.7	280.1	530.2	349.2	964.5	462.4	880.3
	LF/HF	0.36	0.88	0.76	0.41	5.15	0.63	1.21	0.51

43	PSI	38.00	32.40	39.64	26.67	26.15	30.60	45.86	30.98
	TP (ms^2^)	1242.3	1411.1	1271.9	1719.5	1256.2	1819.6	1591.2	1812.5
	VLF (ms^2^)	385.2	344.7	347.0	442.4	412.5	541.0	622.5	525.5
	LF (ms^2^)	252.2	461.2	330.8	472.0	382.2	412.8	446.8	411.8
	HF (ms^2^)	605.0	605.2	594.0	805.1	461.5	865.8	521.9	875.2
	LF/HF	0.43	0.76	0.59	0.60	1.78	0.48	0.80	0.60

44	PSI	87.76	58.74	104.10	75.77	87.02	54.57	63.23	44.89
	TP (ms^2^)	633.8	740.0	1113.2	1418.6	756.6	1485.6	1217.2	1387.6
	VLF (ms^2^)	217.9	251.3	322.8	618.7	297.4	567.3	428.0	372.0
	LF (ms^2^)	249.0	179.8	430.2	347.7	151.4	422.6	529.3	359.4
	HF (ms^2^)	167.0	308.9	360.2	452.2	307.8	495.6	260.0	656.2
	LF/HF	1.30	0.76	1.53	1.14	0.71	0.98	1.63	0.82

45	PSI	47.73	26.60	50.26	33.31	42.95	30.73	70.19	48.99
	TP (ms^2^)	972.6	1753.2	1443.4	1762.9	1439.3	1962.6	1017.7	1518.8
	VLF (ms^2^)	371.9	599.8	495.3	437.2	412.7	763.8	409.6	392.7
	LF (ms^2^)	344.9	458.2	590.4	563.9	632.2	492.8	320.4	643.7
	HF (ms^2^)	255.9	695.2	357.7	761.8	394.4	706.1	287.7	482.4
	LF/HF	1.69	0.69	1.66	0.73	1.71	0.77	1.38	1.32

**Table 2:** The averaged sequence values before massage.

Grey factors	*x*_0_	*x*_1_	*x*_2_	*x*_3_	*x*_4_
Subjects	PSI	TP (ms^2^)	VLF (ms^2^)	LF (ms^2^)	HF (ms^2^)

01	38.09	1797.85	770.11	670.26	357.11
02	82.06	683.10	297.60	179.24	205.64
03	25.86	1685.74	524.09	371.14	790.71
04	42.72	1392.84	561.71	319.80	511.33
05	63.26	1186.25	466.13	428.13	290.50
06	44.21	1156.71	258.54	537.44	360.74
07	67.96	579.73	328.29	163.34	88.09
08	38.55	1325.08	447.01	572.10	305.98
09	32.88	1493.45	635.47	606.76	251.22
10	41.73	1165.58	425.87	280.80	458.92
11	43.30	2102.25	904.75	733.75	463.00
12	119.75	414.51	175.36	108.37	130.02
13	90.69	718.25	200.00	345.25	172.50
14	21.32	4598.75	2090.75	1937.00	571.00
15	54.48	833.96	345.06	242.86	246.05
16	150.57	307.00	100.75	104.25	102.00
17	91.71	1158.88	330.63	415.50	413.00
18	76.32	953.61	405.42	357.57	190.62
19	62.28	1603.33	445.56	571.13	586.61
20	123.84	517.31	185.50	177.13	154.66
21	105.22	566.25	187.68	226.44	152.14
22	56.01	2658.25	1145.38	1141.13	371.75
23	81.53	1257.88	540.06	421.06	296.51
24	67.00	1409.75	552.38	539.50	317.75
25	70.54	2506.25	1133.06	1022.69	350.51
26	38.51	1076.12	322.57	362.34	391.20
27	135.16	360.50	138.06	106.31	116.01
28	35.88	1415.10	615.21	438.54	361.35
29	37.85	1457.39	434.35	594.74	428.31
30	91.20	938.46	265.31	380.38	292.75
31	71.23	2665.93	1153.22	1098.44	414.25
32	36.31	1609.01	561.03	398.01	649.97
33	35.89	1500.08	495.02	573.60	431.46
34	69.68	1021.42	229.55	505.43	286.45
35	48.27	938.61	453.73	332.39	152.49
36	35.02	1852.71	953.15	631.80	267.76
37	32.84	2010.75	560.50	726.75	723.50
38	27.08	3304.75	1325.63	1331.88	647.25
39	121.14	733.08	215.69	259.88	257.50
40	85.39	2775.13	1372.75	1064.63	337.75
41	55.74	1326.50	569.28	473.37	283.85
42	49.28	2201.34	1160.47	690.25	350.62
43	37.41	1340.39	441.80	352.99	545.59
44	85.53	930.23	316.54	339.96	273.74
45	52.78	1218.26	422.38	471.98	323.90

**Table 3:** The averaged sequenced values after massage.

Grey factors	*x*_0_	*x*_1_	*x*_2_	*x*_3_	*x*_4_
Subjects	PSI	TP (ms^2^)	VLF (ms^2^)	LF (ms^2^)	HF (ms^2^)

01	27.26	2145.09	604.52	750.00	803.20
02	57.65	1028.64	289.92	365.22	360.98
03	22.30	2049.12	524.03	568.10	932.14
04	25.36	1596.35	400.63	338.29	857.43
05	46.23	1728.75	582.13	552.13	606.88
06	30.14	2275.88	949.83	617.87	733.18
07	50.69	1067.97	404.82	283.35	404.79
08	26.79	2389.41	801.95	772.93	801.98
09	23.44	2502.94	754.11	952.96	795.85
10	36.24	1446.79	368.57	611.45	466.76
11	31.09	1787.25	718.50	521.25	546.75
12	79.06	610.50	236.25	119.00	255.00
13	70.80	1325.75	465.00	483.00	377.75
14	14.82	1416.00	595.00	331.25	489.25
15	43.47	1257.38	386.70	447.40	435.78
16	104.22	455.50	162.75	196.00	96.75
17	64.10	1681.50	598.88	724.25	358.38
18	51.25	1556.58	495.18	535.98	525.42
19	44.04	2294.51	816.94	988.38	489.19
20	94.19	786.73	271.78	328.06	186.88
21	74.93	968.00	350.63	301.00	316.38
22	42.81	1370.63	530.00	407.13	433.50
23	55.08	1198.38	477.38	320.13	400.88
24	50.94	1556.25	591.75	502.13	462.25
25	41.76	1012.88	415.63	225.13	372.13
26	36.49	1558.62	464.43	425.73	668.46
27	91.64	532.88	199.50	157.50	175.88
28	24.40	2049.65	577.37	645.62	826.64
29	28.20	1801.92	623.67	567.86	597.89
30	67.45	1503.63	531.94	603.63	368.06
31	49.49	1242.25	487.91	395.69	358.41
32	23.83	1822.74	462.33	453.19	907.20
33	25.34	1970.60	705.12	497.23	780.77
34	45.41	1449.10	519.39	433.61	496.11
35	37.07	1797.96	579.47	618.16	600.32
36	27.59	1819.95	537.24	601.09	681.63
37	23.99	2907.50	1035.00	1252.50	620.00
38	19.41	2161.50	815.00	791.88	554.63
39	84.16	1068.50	380.81	460.13	227.56
40	59.52	935.50	378.88	263.63	293.00
41	39.36	1592.55	548.11	461.37	583.06
42	36.86	1715.08	560.81	431.85	722.43
43	30.16	1690.68	463.38	439.46	787.83
44	58.49	1257.92	452.31	327.37	478.24
45	34.91	1749.37	548.38	539.62	661.38

**Table 4:** The results of grey modeling analysis.

Weighting state	TP	VLF	LF	HF
Before	55.1461	55.3678	55.7750	55.1798
After	0.8528	0.5990	0.9215	0.9505

**Table 5:** The results of grey modeling analysis.

Relationship weighting		TP	VLF	LF	HF
*L* = 45	Before	55.1461	55.3678	55.7750	55.1798
	After	0.8528	0.5990	0.9215	0.9505

*L* = 30	Before	56.352	56.118	56.693	56.452
	After	2.8134	2.5409	2.8536	2.9479

*L* = 20	Before	48.066	47.788	48.429	48.2
	After	2.0625	1.8322	2.0638	2.1998

*L* = 15	Before	46.865	46.756	47.059	46.967
	After	0.4175	0.1375	0.4268	0.6119
